# Comprehensive Comparison of the Performance of Autogenous Brachial-Basilic Transposition Arteriovenous Fistula and Prosthetic Forearm Loop Arteriovenous Graft in a Multiethnic Asian Hemodialysis Population

**DOI:** 10.1155/2016/8693278

**Published:** 2016-10-20

**Authors:** Koy Min Chue, Kyi Zin Thant, Hai Dong Luo, Yu Hang Rodney Soh, Pei Ho

**Affiliations:** ^1^University Surgical Cluster, National University Health System, 1E Kent Ridge Road, Singapore 119228; ^2^Department of Cardiac, Thoracic and Vascular Surgery, National University Health System, 1E Kent Ridge Road, Singapore 119228; ^3^Yong Loo Lin School of Medicine, National University of Singapore, 10 Medical Drive, Singapore 117597

## Abstract

*Aim.* For patients who have exhausted cephalic vein arteriovenous fistula (AVF) options, controversy exists on whether brachial-basilic AVF with transposition (BBTAVF) or a forearm arteriovenous graft (AVG) should be the next vascular access of choice. This study compared the outcomes of these two modalities.* Methods.* A retrospective study of 122 Asian multiethnic patients who underwent either a BBTAVF (81) or an AVG (41). Maturation time and intervention rates were analyzed. Functional primary, secondary, and overall patency rates were evaluated.* Results.* The maturation time for BBTAVFs was significantly longer than AVGs. There was also a longer deliberation time before surgeons abandon a failing BBTAVF compared to an AVG. Both functional primary and secondary patency rates were significantly higher in the BBTAVF group at 1-year follow-up: 73.2% versus 34.1% (*p* < 0.001) and 71.8% versus 54.3% (*p* = 0.022), respectively. AVGs also required more interventions to maintain patency. When maturation rates were considered, the overall patency of AVGs was initially superior in the first 25 weeks after creation and then became inferior afterwards.* Conclusion.* BBTAVFs had superior primary and functional patency and required less salvage interventions. The forearm AVG might have a role in patients who require early vascular access due to complications from central venous catheters or with limited life expectancy.

## 1. Introduction

Hemodialysis remains the commonest mode of renal replacement therapy for end stage renal disease patients (ESRD) worldwide. Nearly 80% of ESRD patients in Singapore were on hemodialysis [1]. The Kidney Disease Outcome Quality Initiative (KDOQI) guidelines recommended autogenous arteriovenous radiocephalic (RC AVF) or a brachiocephalic fistula (BC AVF) as the first-line options for vascular access [2]. Yet, due to various reasons, such as either a lack of suitable cephalic veins [3] or failed cephalic vein arteriovenous fistulas (AVF), some patients are unable to achieve hemodialysis via the cephalic vein AVF. For such patients who only have favourable basilic veins over the elbow region, the vascular access strategies will include a brachial-basilic transposition arteriovenous fistula (BBTAVF) or a forearm loop arteriovenous graft (AVG) with either prosthetic or biosynthetic material [2]. Each access type has its advantages and limitations. Although several randomized controlled trials have previously evaluated the patency rates between these 2 different modalities [4–6], they are largely small studies [7] and no consensus conclusion had been reached. Furthermore, few studies addressed such differences in an Asian population.

In this study, we aim to review the performance of the BBTAVF and forearm brachial-basilic AVGs (BB AVGs) of Asian patients with only basilic veins suitable for vascular access in our institution. It is hoped that the results of this study will facilitate clinicians to make an optimal vascular access strategy, thus prolonging the access patency and survival of ESRD patients who are already running out of cephalic vein fistula options.

## 2. Materials and Methods

### 2.1. Patient Selection

A retrospective review of the clinicoradiologic information of 124 patients who underwent either a transposed basilic vein fistula (BBTAVF group) or a forearm loop BB AVG (BB AVG group) procedure between January 2010 to June 2012 at a tertiary referral centre was performed. These patients were all ESRD patients already on hemodialysis who had either no suitable cephalic veins for AVF creation or had previous failed cephalic vein AVF, with a favourable basilic vein identified over the distal arm or elbow region. We define a suitable vein for access creation as a vein more than 2.5 mm measured by ultrasound over the elbow region. The last date of entry of outcomes was November 2013. One patient underwent a preemptive vascular access creation and was excluded. Information collected includes demographic data, comorbidities, and ultrasound measured venous diameter. Outcome assessments include major perioperative morbidity and mortality, access success rate, assisted success rate, functional primary, and secondary and overall patencies of the vascular access. This study obtained Institutional Ethics approval.

All procedures were performed under either local anesthesia with sedation, regional anesthesia, or general anesthesia. Preoperative arterial and venous duplex ultrasound assessments were conducted for all patients and the results showed patients were suitable for both BBTAVF and BB AVG. The actual sites of the brachial artery and basilic vein utilised for anastomosis were determined intraoperatively under ultrasonographic guidance immediately before surgery. The BBTAVF included single-stage and 2-stage procedures [8]. The BB AVG was a forearm loop BB AVG, created using either a synthetic expanded polytetrafluoroethylene (ePTFE) graft (Gore-tex and Gore-Propaten, Gore, AZ, US; Impra, Bard PV, AZ, US) or a biosynthetic graft (Omniflow, LeMaitre, MA, US), based on the individual surgeon's preference. Both arterial and venous anastomoses were performed in an end-to-side manner. There were no changes in the techniques for BBTAVF or BB AVG creation over the study period. The decision to create either a BBTAVF or a BB AVG was made as a consensus between the patient and the primary surgeon, after a thorough discussion of the benefits and limitations of both procedures.

Balloon angioplasty was employed as the salvage technique for failing BBTAVFs and BB AVGs. The decision to intervene was dependent on both clinical as well as dialysis parameters, as described in our previous publication [9]. For thrombosed BB AVGs, a graft thrombectomy followed by angiogram and angioplasty was performed as the salvage procedure. For thrombosed BBTAVFs, balloon angioplasty with or without thrombectomy was performed only if the thrombosis involved a short segment of the fistula. The BBTAVF will be abandoned if there was a long segment thrombosis.

### 2.2. Outcome Definitions

Three patency rates were sorted: (1) functional primary patency, defined as the time from established successful access cannulation until the time where any intervention aimed to maintain or reestablish access patency [10]; (2) functional secondary patency, defined as the time from established successful access cannulation until the time where the access has to be abandoned or the patient has demised [10] (the functional primary and secondary patencies are only applied to vascular accesses matured for successful cannulation); and (3) overall patency, defined as the patency of all studied hemodialysis accesses. This included accesses with nonsalvageable primary failure. The overall patency is assigned to be zero if the access has failed primarily. To enable uniform data capture, the date of established successful access cannulation was recorded as the date where any temporary central venous hemodialysis catheter was removed or the date of successful usage of the access for hemodialysis if no bridging temporary catheter was required.

### 2.3. Statistical Analysis

Chi-square analysis was performed to identify intergroup differences in patient demographics. Patency rates of the hemodialysis access were calculated with the Kaplan-Meier survival analysis. A log-rank test was used to compare the differences in patency rates between the two groups. For continuous variables which were not normally distributed, Kruskal-Wallis 1-way ANOVA test was performed. A *p* value of less than 0.05 was considered statistically significant. All data analysis was performed via IBM Statistical Product and Service Solutions (SPSS) version 21 (PASW Statistics 21.0).

## 3. Results

During the study period, a total of 123 patients underwent either a BBTAVF or forearm BB AVG procedure. Eighty-two patients underwent BBTAVF (either single-staged or two-staged) procedure, while 41 patients underwent a forearm loop BB AVG creation. The average follow-up period for BBTAVFs was 100 + 35.0 (6–191) weeks and for BB AVG was 116 + 42.0 (14–186) weeks. Out of these 82 BBTAVF patients, there was one unrelated 30-day mortality and this was excluded. In total, 59 (72.8%) out of 81 BBTAVFs were created as a 2-staged procedure and the rest as a single-staged procedure. In the BB AVG group, ePTFE grafts were used in 80.5% (33/41) patients and 19.5% (8/41) patients received biosynthetic grafts.

The mean age for the BBTAVF and BB AVG groups was comparable. Majority of them had hypertension (87.7% in BBTAVF; 87.8% in BB AVG), diabetes mellitus (61.7% in BBTAVF; 56.1% in BB AVG), and hyperlipidemia (46.9% in BBTAVF; 46.3% in BB AVG). Most common cause of ESRD was diabetes mellitus. No statistically significant differences were detected between the 2 groups in terms of their demographics (Table 1). Majority of the vascular accesses were created on the left side (76.2%, 93/122), reflecting the preference for access creation on the nondominant arm. No statistically significant differences in the preoperative venous diameter were found between the BBTAVF and BB AVG groups (*p* = 0.118) and also between single-staged (3.4 ± 1.0 mm) and two-staged (3.15 ± 1.0) BBTAVFs (*p* = 0.231). In our study, the presence of a previous dialysis catheter did not significantly impact the decision on the type of vascular access. Up to 80.5% (66/82) of the BBTAVF group and 90.2% (37/41) of the BB AVG group had a dialysis catheter in situ prior to access creation. The mean length of time from catheter insertion to creation of a vascular access in the BBTAVF and BB AVG groups was 68.6 and 81.9 weeks, respectively (*p* = 0.416). The mean number of previous catheter exchanges also did not seem to impact the surgeons' decision for the type of vascular access (0.87 exchanges in BBTAVF; 0.92 exchanges in BB AVG, *p* = 0.874).

### 3.1. Primary Failure and Assisted Success

In total, 23 of the 81 (28.4%) BBTAVFs and 8 of the 41 (19.5%) BB AVGs created had nonsalvageable primary failure. Within the 23 failed BBTAVFs, 31.8% (7/22) were single-staged and 27.1% (16/59) were intended as two-staged procedures. Majority of those intended as two-staged procedures (87.5%, 14/16) failed after the 1st stage (Table 2). The most common cause of primary failure for both BBTAVFs and BB AVFs were due to complete access occlusion and loss of flow, which occurred in 78.3% (18/23) and 87.5% (7/8), respectively. There were 17.4% (4/23) of the BBTAVFs which were patent but had problems associated with cannulation or upper limb swelling which necessitated access abandonment. The remaining 4.3% (1/23) of the BBTAVFs failed to mature to allow for adequate cannulation. One patient in the BB AVG group demised prior to successful graft cannulation.

Interestingly, for all accesses with primary failure, there appeared to be a longer deliberation period from time of access creation to access abandonment in the BBTAVF group (12.1 + 10.4 weeks) compared to the BB AVG group (6.5 + 6.4 weeks), though it did not reach statistical significance (*p* = 0.155). Similarly, the time interval from creation of the index access to a subsequent new access creation also seemed to be longer in the BBTAVF group (22.5 ± 18.5 weeks) compared to the BB AVG group (10.3 ± 6.6 weeks), though it again did not reach statistical significance (*p* = 0.131) (Table 3).

### 3.2. Accesses Successfully Used for Hemodialysis and Time to Maturation

Three patients (3.7%) in the BBTAVF group and 8 patients (19.5%) in the BB AVG group required assistive interventions after access creation before the access was successfully used for hemodialysis. For the BBTAVF group, 1 had early central vein stenosis requiring an angioplasty 9 days after access creation, while the other 2 patients had a fistuloplasty for failure of maturation 3 months after creation. For the BB AVG group, early graft thrombosis (within postoperative day 1) occurred in 4 patients and required graft thrombectomy with or without revision of anastomosis. One patient had significant steal syndrome requiring arterial bypass. One patient had an angioplasty procedure done for high venous pressures and arm swelling 1 month after operation. The remaining 2 patients had a venogram procedure done but with no interventions, all within 40 days postoperatively.

There were no statistical significant differences between the success rates in both groups (71.6% for BBTAVFs and 80.5% for BB AVGs, *p* = 0.287). Mean maturation time was significantly longer in the BBTAVF group, 17.7 (±18.0) weeks, compared to 6.0 (±5.4) weeks in the BB AVG group (*p* < 0.001).

### 3.3. Functional Primary and Secondary Patency Rates of Successful Accesses

**Figure 1:**
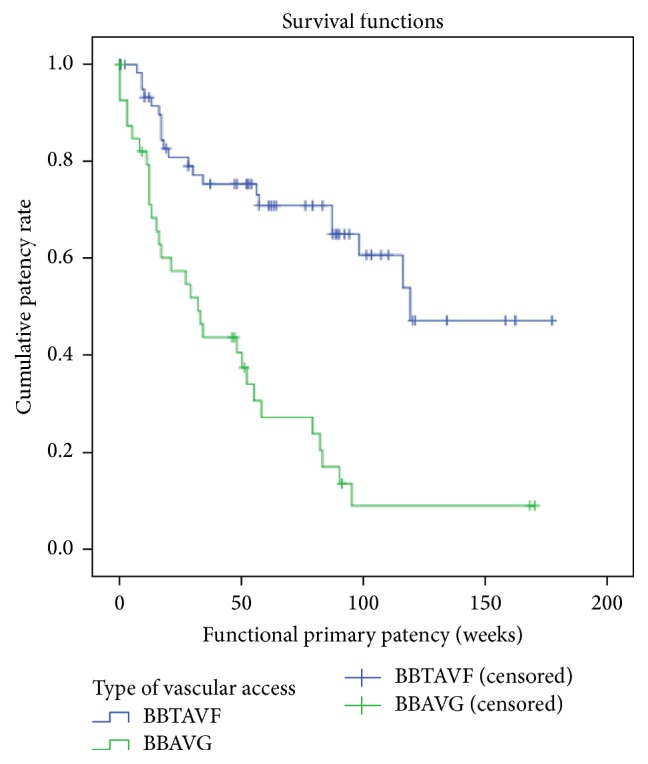
Functional primary patency (weeks) of BBTAVF and forearm loop BB AVG. Cumulative primary functional patency rates at 1 year for BBTAVF: 73.2%. Cumulative primary functional patency rates at 1 year for BB AVG: 34.1% (*p* value < 0.001).

**Figure 2:**
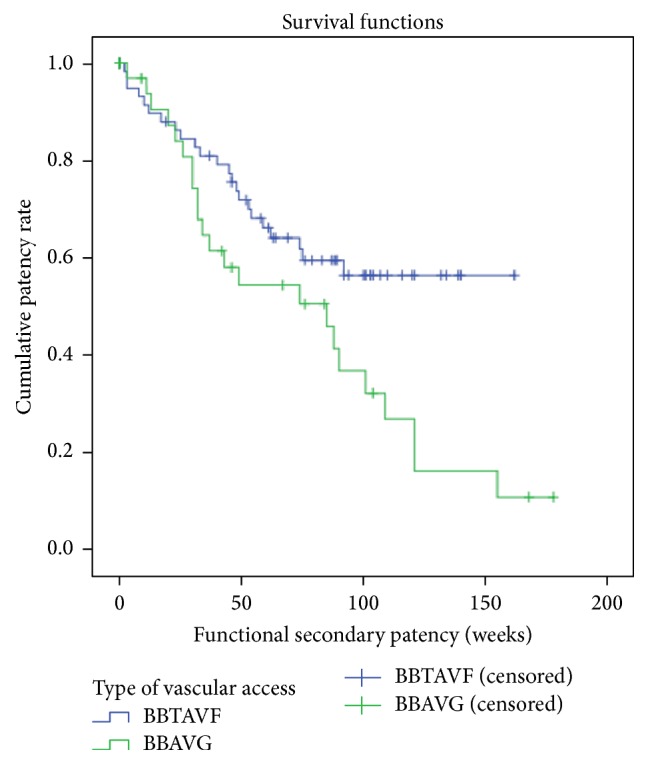
Functional secondary patency (weeks) of BBTAVF and forearm loop BB AVG. Cumulative functional secondary patency rates at 1 year for BBTAVF: 71.8%. Cumulative functional secondary patency rates at 1 year for BB AVG: 54.3% (*p* value: 0.022).

A statistically significant lower functional primary patency rate was observed for the BB AVG group compared to the BBTAVF group, with a cumulative 1-year functional primary patency rate of 73.2% and 34.1% for BBTAVF and BB AVG groups, respectively (*p* < 0.001; Figure 1). A similar trend was observed for functional secondary patency rates as well, with a cumulative 1-year functional secondary patency rate of 71.8% and 54.3% for BBTAVFs and BB AVGs, respectively (*p* = 0.022; Figure 2).

### 3.4. Overall Patency Rates for All Created Accesses

**Figure 3:**
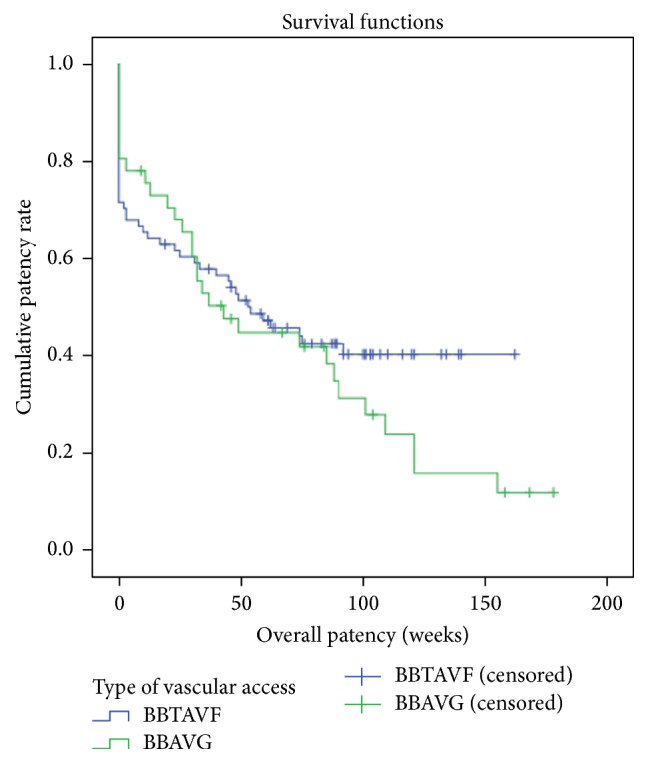
Overall patency (weeks) for BBTAVF and forearm loop BB AVG, taking into account accesses which were never usable due to primary failure. Cumulative overall patency rates at 1 year for BBTAVF: 40.3%. Cumulative overall patency rates at 1 year for BB AVG: 12.0%. (*p* value = 0.291).

When vascular accesses with primary failure were taken into evaluation of the overall patency, there was a higher patency rate for the BB AVG group compared to the BBTAVF group in the initial 25 weeks. Subsequently, the 2 patency tracings crossed. After 30 weeks, the BBTAVF group had a better overall patency rate compared to the BB AVG group (Figure 3).

### 3.5. Total Number of Salvage Procedures Required

The mean number of salvage interventions to maintain patency after maturation and successful cannulation was significantly higher in the BB AVG group (averaged 2.2 ± 1.2 interventions for BB AVG group, 1.5 ± 1.0 interventions for BBTAVF group, resp., *p* = 0.043). However, there were no statistically significant differences in the number of surgical thrombectomies performed for both groups (1.0 ± 0 thrombectomies for BBTAVF group and 1.5 ± 0.8 in the BB AVG group, resp., *p* = 0.476).

### 3.6. Secondary Autogenous Arteriovenous Fistula

One of the purported theoretical advantages for a forearm BB AVG creation was the possibility of it being an interim procedure to allow arterialisation of the outflow basilic vein, for a subsequent Type 1 secondary AVF creation [11–13] in the future. Yet, in our series, only 1 patient (2.4%) in the BB AVG group eventually had a suitable venous anatomy for a secondary BBTAVF procedure after her initial BB AVG failed. She had an initial basilic vein diameter of 3.4 mm, which subsequently increased to 6 mm by the time a secondary BBTAVF was created using the outflow basilic vein.

### 3.7. Crossover Patients

There were a total of 5 patients in the BB AVG group with primary failure due to graft thrombosis who subsequently had an AVF created. All of the BB AVGs were never cannulated. Three of them had an ipsilateral arm BBTAVF created, while 2 of them had an AVF created on the contralateral arm.

There were 4 patients in the BBTAVF group with primary failure of their fistula who eventually required a BB AVG creation. Amongst these patients, only 1 of them had a functional BBTAVF but failed to be cannulated due to upper limb edema. This access was subsequently converted to a BB AVG created on the contralateral side after 8 months. All the other 3 patients had primary thrombosis of the initial BBTAVF and underwent an upper arm BB AVG creation using the proximal outflow vein of the previous BBTAVF.

## 4. Discussion

After exhausting the primary vascular access options of both RC and BC AVFs, there is still controversy on the next optimal type of secondary or tertiary vascular access procedure [6, 7, 14]. Current recommendations are for either an autogenous BBTAVF or a forearm loop BB AVG. Several studies have favoured the BBTAVF over the forearm loop BB AVG procedure on the basis of better patency rates and fewer interventions [5, 6, 15]. However, these studies report only the outcomes from accesses that were successful initially and primary failure cases were excluded from the analysis. Therefore, this might result in an overestimation of the successes of BBTAVFs compared to BB AVGs, as they failed to take into account issues with access creation and maturation.

In our series, the primary failure rate of BBTAVFs (28.4%) was comparable with published data from a review by Dix Jr. et al., between 0 and 38% [16]. Though not statistically significant (*p* = 0.287), the primary failure rate of the BB AVG group (19.5%) was obviously lower than that of the BBTAVFs. Furthermore, the maturation time for BB AVGs was significantly shorter than that of BBTAVFs [5, 6, 17, 18]. When factors like maturation time and primary failure are taken into account, for the first 25 weeks, the forearm loop BB AVG was superior in terms of overall access patency compared to the BBTAVFs. Subsequently, the BBTAVF group showed a more favourable overall patency rate (Figure 3). We thus propose that the primary success rate and maturation time should be taken into evaluation in any future randomized studies of these 2 access strategies.

Proponents of AVGs have suggested that, in patients with a limited life expectancy, an AVG can be considered over an AVF as the preferred vascular access [7, 19]. Though our findings echoed these suggestions given the initial superiority of the BB AVGs over the BBTAVFs, we believe the decision to create a BB AVG over a BBTAVF should still be made on a case-by-case basis. In our series, even in elderly patients, the decision for a BBTAVF versus a BB AVG was made between the surgeon and the patient, sometimes with their family members as well, after a thorough explanation of the benefits and limitations of both access modalities. Given that the Kaplan-Meier curve approached equivalence for both the BBTAVF and BB AVG group at about 25 weeks, we argue that, in patients who can tolerate a longer duration of tunnelled central venous catheter in situ, it might be better to persist with the catheter and proceed with a BBTAVF rather than the BB AVG, as the benefits of the BB AVG seemed to be short-lived. However, we do acknowledge that this must be balanced with the increased risk of adverse events related to prolonged central venous catheterisation and the impaired quality of life associated with the tunnelled catheter. Furthermore, the average survival of hemodialysis dependent elderly patients has also improved over the years, to an average of 2 years or more [20, 21]. Thus, only the small proportion of ESRD patients, who either have a very limited life expectancy or are prone to develop tunnelled central venous catheter complications, are more likely to benefit from a BB AVG than a BBTAVF. For the remaining majority, BBTAVF should be considered as the better option for those without cephalic vein AVF options.

For nonmaturing vascular accesses, though not statistically significant, our study suggested that clinicians might take a longer time to decide abandonment of a BBTAVF (12.1 weeks) compared to a BB AVG (6.5 weeks) for another access creation. This longer deliberation period seemed to translate into a longer time interval before a subsequent access creation as well for a BBTAVF (22.5 weeks) compared to a BB AVG (10.3 weeks). We believe the decision-making process of access abandonment could be an area of future research, as the time taken to wait out on a BBTAVF to mature should be balanced with the risks of central catheter related complications. This is even more relevant considering that these patients might have underlying suboptimal venous anatomy which rendered them unsuitable for cephalic vein AVFs in the first place, and the presence of a tunnelled catheter is a risk factor for nonmaturation as well [22].

More interventions were needed to maintain the patency of the BB AVGs, echoing previous findings from other studies [5, 7]. However, the early thrombosis rate of the BB AVGs in our series was 24.3% (10/41), which was higher than the reported 6.7 to 15.7% [5, 15]. We attempted to explore if the graft material played a role in early AVG thrombosis. The subgroup analysis, however, did not show any statistically significant differences in early thrombosis rate between standard ePTFE versus biosynthetic grafts (*p* = 0.653, data not shown). In total, 10 patients with BB AVG had early thrombosis requiring thrombectomy. 4 of them were successfully salvaged, all of them being synthetic ePTFE grafts. 6 cases were unsalvageable, 1 of them being a biosynthetic graft. Clinical decision of arterial inflow and venous outflow selection and surgical technique probably played a role in early acute thrombosis.

Where secondary AVFs are concerned, only 1 patient in our series eventually had a secondary AVF created using an arterialised outflow vein of a previously constructed BB AVG. We hypothesize that the low rates of secondary AVF creation in the BB AVG group could be due to juxta-anastomotic stenosis or thrombosis extending proximally into the native vein, rendering the proximal basilic vein eventually also unsuitable for BBTAVF creation. The barotrauma to the surrounding native basilic vein from repeated salvage angioplasty might also be a contributory factor resulting in stenosis of the outflow vein.

One limitation of our study is the small sample size and also a smaller representation for the forearm BB AVG group. This could be because of our institution's keen adoption of the fistula first initiative for all patients. Another limitation of this study is its retrospective nature and hence naturally prone information bias, as the accuracy of the analysis is dependent on the meticulous recording and storage of data. However, given the paucity of Asian data reporting outcomes of BBTAVFs versus BB AVGs, we hope that the information provided by this study will shed some light into the choice of basilic vein vascular accesses in this group of patients and also be of value for future meta-analysis studies.

## 5. Conclusion

In this retrospective study of Asian multiethnic hemodialysis patients who have exhausted cephalic vein AVF options, compared to BB AVG, BBTAVFs had better primary and functional patencies and required significantly less salvage interventions. However, the more lengthy maturation time and seemingly higher rates of primary failure would suggest that the BB AVG still has a role to play in patients who require early vascular access availability due to complications from central venous catheters or with limited life expectancy.

## Figures and Tables

**Table 1:** Demographics and characteristics of the population from January 2010 to June 2012.

	BBTAVF	BB AVG	*p* value
Age (years)	59.14	57.24	0.449
Gender			
Male	49 (60.5%)	23 (56.1%)	0.641
Female	32 (39.5%)	18 (43.9%)	
Hypertension	71 (87.7%)	36 (87.8%)	0.981
Diabetes mellitus	50 (61.7%)	23 (56.1%)	0.549
Hyperlipidemia	38 (46.9%)	19 (46.3%)	0.952
Ischemic heart disease	21 (25.9%)	13 (31.7%)	0.501
Cause of ESRD			
Diabetic nephropathy	48 (59.3%)	21 (51.2%)	0.126
Primary glomerulonephritis	2 (2.5%)	3 (7.3%)	
Autoimmune glomerulonephritis/disease	5 (6.2%)	1 (2.4%)	
Hypertension and renovascular disease	8 (9.9%)	0 (0%)	
Polycystic kidney disease/other cystic diseases	3 (3.7%)	4 (9.8%)	
Vesicoureteric reflux/chronic pyelonephritis	1 (1.2%)	1 (2.4%)	
Obstructive stone disease	1 (1.2%)	3 (7.3%)	
Miscellaneous	1 (1.2%)	0 (0%)	
Unknown	12 (14.8%)	8 (19.5%)	

**Table 2:** Outcomes of the vascular access after creation in the BBTAVF and BB AVG groups.

	BBTAVF, *n* (%)			BB AVG, *n* (%)
	1-staged	2-staged	Total	
Primary success	14 (63.6%)	41 (69.5%)	55 (67.9%)	25 (61%)
Intervention-aided success	1 (4.6%)	2 (3.4%)	3 (3.7%)	8 (19.5%)
Primary failure	7 (31.8%)	16 (27.1%)	23 (28.4%)	8 (19.5%)

**Table 3:** Maturation and interventions for the vascular access in the BBTAVF and BB AVG groups.

	BBTAVF	BB AVG	*p* value
Mean venous diameter (mm)	3.2 (±1.0)	2.9 (±0.7)	0.118
Mean maturation time (weeks)	17.7 (±18.0)	6.0 (±5.4)	**0.000**
Mean number of interventions (*n*) to maintain patency	1.5 (±1.0)	2.2 (±1.2)	**0.043**
Mean number of surgical thrombectomies (*n*)	1.0 (±0.0)	1.5 (±0.8)	0.476
For failed accesses, mean time from last creation to decision of abandonment (weeks)	12.1 (±10.1)	6.5 (±6.4)	0.155
For failed accesses, mean time from access creation to next access creation (weeks)	22.5 (±18.5)	10.3 (±6.6)	0.131
